# Nervous Reflexes of Nasal Origin

**Published:** 1890-03

**Authors:** 


### Abstracts.

---

### NERVOUS REFLEXES OF NASAL ORIGIN.

In the *Revue de Laryngologie d'Otologie et de Rhinologie* for January, 1890, Dr. Eli Goris, of Brussels, presents his conclusions concerning the nervous reflexes of nasal origin. These conclusions are based upon the study of eleven cases, which are given in full. The reflexes observed were asthma, migraine, neuralgia of various parts of the head, paralysis of the eyelid, and even melancholia with suicidal tendency. The nasal lesions upon which these depended were hypertrophies, adenoid vegetations, and mucous polyps, and occasionally simple dilation of the so-called erectile tissues. Upon removing these lesions, the symptoms, as just given, disappeared. He presents the results of his studies as follows:

1. The same reflex may depend upon nasal lesions of the most diverse histology.

2. These lesions may be found in any part of the cavities of the nose and naso-pharynx.

3. The pathology of the reflex symptoms of nasal origin can be entirely explained by the connection between the trigeminus and the other centers of innervation.

---

Cod Liver Oil for Children.—Dr. W. F. Waugh, in the *Dietetic Gazette*, gives the following mode of the administration of cod liver oil to children: "I have made it a rule to prescribe cod liver oil for children, mixed with an equal quantity of orange syrup, and with each renewal diminished the proportion of the syrup until it was omitted entirely. I do not now recollect ever to have known a child to refuse this mixture, while they rarely fail to acquire a taste for the oil itself.

